# Sleep Characteristics and Influencing Factors of Sleep Quality in Patients With Inflammatory Bowel Disease-Peripheral Arthritis

**DOI:** 10.3389/fmed.2019.00190

**Published:** 2020-01-06

**Authors:** Yin Zhang, Bin Pi, Xianlin Xu, Ying Li, Xiangfan Chen, Ningxi Yang, Xiaoyan Li

**Affiliations:** ^1^Department of Gastroenterology, Huiyang Sanhe Hospital, Huizhou, China; ^2^Department of Orthopedics, Fifth Affiliated Hospital, Guangzhou Medical University, Guangzhou, China; ^3^School of Health Science, Wuhan University, Wuhan, China; ^4^College of Humanities and Social Sciences, Harbin Engineering University, Harbin, China

**Keywords:** inflammatory bowel disease, arthritis, sleep disturbances, polysomnography, pittsburgh sleep quality scale (PSQI)

## Abstract

**Background:** Patients with inflammatory bowel disease-peripheral arthritis (IBD-PA) often accompany by sleep problems, but few studies have used polysomnography to investigate the objective sleep characteristics of IBD-PA.

**Methods:** Patients in IBD-PA group, inflammatory bowel disease (IBD) group, healthy control (HC) group were examined by polysomnography (PSG) and the sleep characteristics were compared. PSG and Pittsburgh Sleep Quality Scale (PSQI) indices were compared between active and remission IBD-PA patients. The correlation between disease activity and sleep quality in IBD-PA patients was analyzed. The influencing factors of sleep efficiency of IBD-PA patients were analyzed.

**Results:** The total sleep time (TST), rapid-eye-movement sleep (REM) time, slow wave sleep (S3 + S4) and sleep efficiency (SE) in the IBD-PA group were significantly less than those in the HC group and IBD group (*P* < 0.05), while the number and time of wake after sleep onset (WASO) and sleep latency (SL) were significantly longer than those in the HC group and IBD group (*P* < 0.05). To IBD-PA patients, the disease activity was negatively related to sleep quality. There was a significant difference in SE, the number of WASO, S1, S3+S4 of PSG, as well as the PSQI total score between active and remission patients. Abdominal pain, joint pain, depression, and C-reactive protein were the influencing factors of sleep efficiency.

**Conclusions:** The sleep quality of patients with IBD was poor than the control group, and that of patients with IBD-PA was even worse. Therefore, sleep management should be included in IBD management.

## Introduction

Inflammatory bowel diseases (IBD) include ulcerative colitis (UC) and Crohn's disease. IBD is a non-specific, recurrent intestinal inflammatory disease involving all segments of the digestive tract (1). Inflammatory bowel disease-peripheral arthritis (IBD-PA) is an arthritis associated with IBD, which is the most common parenteral manifestation of IBD. About 15–20% of patients with IBD may have arthralgia or arthritis. IBD-PA belongs to reactive arthritis and the diseases involve peripheral joint and axial joint. It can involve not only peripheral joints which leads to pain and swelling in knee, ankle, shoulder, elbow, and wrist joints, but also may involve medial axis joints, which can cause sacroiliac arthritis and hip joint involvement. A few patients can develop ankylosing spondylitis. Among them, peripheral arthritis is the most common, and major joints of lower limbs such as knee, ankle, and foot are the most common involve (2–4).

For patients with IBD, they not only suffer physical discomfort, but also often accompanied by depression, anxiety, pain, sleep disorders, and other mental and psychological problems (5–7). Good sleep is a necessary condition for maintaining good health. Previous studies have found that patients with IBD have a higher incidence of sleep disorders (8). For patients with IBD-PA, they often face more serious sleep problems because of joint symptoms.

Sleep plays an important role in patients with IBD. Previous studies have confirmed that good sleep can help reduce fatigue, improve quality of life and alleviate gastrointestinal symptoms in patients with IBD (9, 10). In IBD patients, the relationship between sleep, and mucosal activity and recurrence has been confirmed (11, 12). Therefore, it is very important to know the sleep status of patients, which will help provide the basis for further sleep intervention in the future, and help patients improve the treatment effect ultimately. Because of the high incidence of the disease in Western countries, the psychological and behavioral research of patients is relatively mature. In recent years, the incidence of IBD has increased in China and other Asian countries, but the related psychiatric research is less (13). Most of the previous studies used scales such as Pittsburgh Sleep Quality Scale (PSQI) to measure subjective sleep quality in patients with IBD (14). They confirmed IBD patients suffered poor sleep quality. For example, Sisson reported the mean PSQI was 9.6 in IBD patients (15). Bucci found the 41.4% of CD patients and 31.6% of UC patients reported the PSQI score over 5 (9). In a study involving 120 Chinese patients, researchers found their average PSQI score was 8.07 ± 2.91. Specifically, the sleep subjective quality score was 1.54 ± 0.66, the sleep latency score was 1.62 ± 0.74, the sleep duration score was 1.16 ± 0.71, the habitual sleep efficiency score was 0.89 ± 0.63, the sleep disturbances score was 1.10 ± 0.42, the use of sleeping medications score was 0.17 ± 0.37, and the daytime dysfunction score was 1.59 ± 0.76 (16). Although several researches have reported the sleep status of patients with IBD, the study of patients with IBD-PA and research on objective sleep quality are very limited. As an objective tool, PSG plays an important role in understanding the sleep parameters and structure of patients. In this study, sleep characteristics of Chinese patients with IBD-PA and IBD were measured by both polysomnography (PSG) and PSQI. Then the influencing factors of sleep quality of patients with IBD-PA were analyzed.

## Materials and Methods

In this study, the sample size of this study was calculated according to the following sample size formula:

$$n=\frac{\lambda }{2{\left[\sin^{-1}{\left(p_{\max}\right)}^{1/2}-\sin^{-1}{\left(p_{\min}\right)}^{1/2}\right]}^{2}}$$

According to the former literature (17), p_max_ = 0.82, p_min_ = 0.51, α = 0.05, β = 0.1, ν = *k*-1 = 3–1 = 2, λ_0.05, 0.1;2_ = 12.65. The results was *n* = 87 in each group. Therefore, between June 2018 and July 2019, 120 patients with IBD and 120 patients with IBD-PA were recruited through snowball and online recruitment, and 120 healthy persons were selected as control group. The patients met the predetermined criteria for eligibility: (1) Each patient was diagnosed with IBD or IBD-PA. For IBD-PA, patients with peripheral arthritis were enrolled. They knew their diagnosis and were able to provide informed consent and complete all questionnaires with full awareness of their diagnosis. (2) Each patient was age between 18 and 70. (3) Each patient had clear thinking and no cognitive impairment. (4) They were being hospitalized and the prescription may be mercaptopurine/azathioprine and/or mesalamine according to their condition. Exclusion criteria were as follows: Patients who suffer from other diseases that may affect the evaluation of the scale, such as other joint diseases, skin itching, cancer, severe infection, acute cardiovascular and cerebrovascular diseases, encephalopathy such as Parkinson's and epilepsy, schizophrenia and other mental diseases, severe respiratory, and liver and kidney diseases affecting sleep, prostate diseases causing frequent nocturia, etc. The exclusion criteria of the control group were the same as those of the patients with IBD.

### Procedures

Firstly, PSG was used to monitor the sleep of three groups for two nights, and the data of the second night were analyzed to minimize the changes of sleep habits caused by environmental changes. At the same time, three groups of subjects were surveyed by PSQI questionnaire. Then the PSG parameters and PSQI scores of the three groups were compared. PSG and PSQI indices were compared between active and remission IBD-PA patients. The correlation between disease activity and sleep quality in IBD-PA patients was analyzed. Finally, because sleep efficiency is an important indicator of sleep quality, so we set it as the dependent variable, and analyzed the influencing factors of it.

### Measures

#### Essential Information

In the questionnaire, we designed the essential information section. This section included sociol-demographic information, such as age, sex, marital status, educational background, working situation, etc.

#### Disease and Treatment Condition

For the patient group, disease activity, disease type, course of disease, medication, perianal disease, disease extension, and nocturnal gastrointestinal symptoms were investigated. The diagnosis of IBD or IBD-PA was verified through chart review, which was provided by the patients.

#### Depressive Mood

Zung's self-rating depression scale (SDS) was used to assess patients' depressed mood. The SDS scale is a 20 point self-assessment scale used for screening for depressive mood in 1 week. Participants are asked to choose their option response for each question. The total score of the 20 items was SDS raw score. Multiply raw score by 1.25 and keep the integer part, the standard score is calculated. Higher standard scores indicate severer depression (18). According to the norm of China, the standard score in 50 points and above prompts depressive mood.

#### Joint Pain

The degree of joint pain in patients with IBD-PA was measured by numerical rating scale (NRS). Pain intensity was described with 0–10. Higher numbers indicate a stronger sense of pain (19).

#### Abdominal Pain

Patients provided ratings of abdominal pain with a 4-point Likert scale (i.e., none, mild, moderate, or severe). The higher the scores means the degree of the pain is higher (the score ranges from 1 to 4).

#### C-Reactive Protein

C-reactive protein was measured by latex agglutination turbidimetry. The patient was extracted 3 ml venous blood on an empty stomach in the morning and monitored after serum separation. The reference value is 0–10 mg/L.

#### Disease Activity

Harvey–Bradshaw for Crohn's disease (CD) and the Mayo Endoscopy Score (MES) for ulcerative colitis (UC) were used to measure the disease activity of the patients. Harvey-Bradshaw scale for CD is composed of 5 parameters: general well-being, abdominal pain, number of liquid stools per day, abdominal mass, and complications (20). In this scale higher scores indicates the disease activity is more active. Patients scores 4 or less indicates the remission stage, while scores more than 4 indicates the active stage. Mayo Endoscopy Score consists of diarrhea, rectal bleeding, enteroscopy, and physician evaluation. It ranges from 0 to 3: 0 for inactive diseases, 1 for mild stage, 2 for moderate stage, and 3 for severe stage (21).

#### Sleep

Sleep monitoring was performed in three groups by polysomnography. All patients were monitored by PSG throughout the night (at least 7 h). According to the international standard method, the surface disc electrodes were used to record the electroencephalogram (EEG) signals of six parts synchronously (F3-A2, F4-A1, C3-A2, 01-A2, 02-A1). Two surface electrodes were taken to record the electromyogram (EMG) of the chin. An electrode was placed at 1 cm above and below the lateral canthus to record the eye movements of the left and right eyes. The impedance thoracoabdominal mobility transducer was used to record the patients' chest and abdominal respiratory mobility. Pressure sensors placed in the nasal vestibule were used to record oronasal respiratory airflow. Sleep parameters are as follows: (1) Total sleep time (TST):total real sleep time during night sleep monitoring. (2) Sleep latency (SL): the time from light off to the first sleep period begins. If the SL>30 min, patients were defined as difficulty falling asleep. (3) Sleep efficiency (SE):ratio of TST to bedtime. (4) Rapid-eye-movement sleep (REM) time: total time of REM during nighttime sleep. (5) Times of wake after sleep onset (WASO): number of awakenings from sleep to the end of sleep with each time not <15 seconds. (6) Total time of WASO: total time of awakenings from sleep to the end of sleep. In addition, sleep structure is divided into four stages (I-IV).

Pittsburgh sleep quality index (PSQI) was used to evaluate the subjective sleep quality of patients in the past month. Eight items of the scale consist of seven parts, including sleep quality, sleep latency, sleep duration, habitual sleep efficiency, sleep disturbances, use of sleeping medications and daytime dysfunction. Each part is scored in 0–3 grades. The cumulative score of each component is the total score of PSQI. The higher the score, the worse the sleep quality (22).

### Statistical Analyses

Epi Info version 3.1 (Centers for Disease Control and Prevention, Atlanta, GA, USA) was used for the entry and summary of the data. IBM SPSS Statistics, version 20 (IBM Corp., Armonk, NY, USA) was used to analyze the data. All data are expressed as mean values the standard deviation. Differences in frequencies among groups were calculated using χ^2^ test. One-way analysis of variance (ANOVA) with a least significant difference (LSD) test was used to compare the three groups on the indices. Pearson's correlation analysis was used to measure the correlation between disease activity and sleep quality in IBD-PA patients. The PSG and PSQI indices of active and remission IBD-PA patients were compared by *t* test. Univariate analysis was used to compare sleep efficiency of patients with different demographic characteristics and disease conditions. Multivariate linear regression was used to evaluate the influencing factors of sleep efficiency.

## Results

### Sample

Four hundred twelve people received our invitation and 360 people took part in the research with a participation rate of 87.38%. There was no significant difference in basic demographic and clinical characteristics among the three groups. The results are summarized in Table 1.

**Table 1:** Comparison of basic demographic and clinical characteristics of 13 groups of subjects.

	**HC**	**IBD**	**IBD-PA**	***t/F/χ^2^***	***P***
	***n* = 120**	***n* = 120**	***n* = 120**		
Age: mean (± SD)	36.28 ± 5.12	36.01 ± 5.88	35.55 ± 6.96	0.45	0.64
Sex: Males, *N* (%)	65, 54.17%	61, 50.83%	64, 53.33%	0.29	0.87
Course of disease: under 5 years *N* (%)	/	59, 49.17%	59, 49.17%	1.35	0.24
Disease species: crohn's disease *N* (%)	/	39, 32.5%	33, 27.5%	0.71	0.40
Disease activity: active stage *N* (%)	/	42, 35%	39, 32.5%	0.17	0.68

### Comparison of PSG Indices of the Three Groups

The PSG data of three groups were analyzed by variance analysis and LSD test. Variance analysis results showed that there were significant differences in TST, sleep latency, sleep efficiency, the number and time of WASO and sleep structure among the three groups (*P* < 0.001). The results are given in Table 2.

**Table 2:** Variance analysis results of the differences between IBD group, IBD-PA group and control group in PSG sleep parameters.

**PSG indexes**	**Control group**	**IBD group**	**IBD-PA group**	***F***	***P***
	***n* = 120**	***n* = 120**	***n* = 120**		
TST (min)	430.58 ± 20.01	378.29 ± 23.85	341.88 ± 16.85	568.83	<0.001
SL (min)	21.38 ± 5.19	34.49 ± 6.26	39.93 ± 12.01	155.70	<0.001
SE (%)	85.45 ± 4.89	76.45 ± 6.07	69.66 ± 8.27	174.61	<0.001
Number of WASO	0.95 ± 1.11	1.92 ± 0.42	3.34 ± 0.87	240.49	<0.001
Time of WASO	11.75 ± 12.11	36.62 ± 31.06	50.67 ± 10.14	519.45	<0.001
REM (min)	85.21 ± 5.24	65.62 ± 7.38	57.42 ± 7.96	505.37	<0.001
S1	21.52 ± 4.13	39.64 ± 5.48	35.78 ± 6.04	392.18	<0.001
S2	215.61 ± 15.94	204.99 ± 18.10	200.94 ± 11.10	29.29	<0.001
S3 + S4	108.24 ± 10.22	68.04 ± 19.99	47.74 ± 9.82	568.28	<0.001

LSD test results were showed in Table 3. Compared with the control group, the indexes of IBD group and IBD-PA group were significantly different (*P* < 0.001). In addition, there were significant differences between IBD-PA group and IBD group (*P* < 0.001). Overall, the sleep quality of patients with IBD and IBD-PA was lower than healthy control group, and the sleep quality of patients with IBD-PA was worse lower than IBD group. In detail, the TST, REM time, slow wave sleep (S3 + S4) and sleep efficiency (SE) in the IBD and IBD-PA group were significantly less than those in the HC group (*P* < 0.001). In terms of the above parameters, IBD-PA group were significantly less than those in the IBD group (*P* < 0.001). By contraries, compared with the HC group, the number and time of WASO and sleep latency (SL) in IBD group and IBD-PA group were significantly longer (*P* < 0.001). In terms of the above parameters, IBD-PA group were significantly longer than those in the IBD group (*P* < 0.001).

**Table 3:** LSD test results of the differences between IBD group, IBD-PA group and control group in PSG sleep parameters.

**PSG indexes**	**Group**	**Mean deviation**	**SD**	***P***
TST (min)	Control vs. IBD	52.26	2.63	<0.001
	Control vs. IBD-PA	88.38		<0.001
	IBD vs. IBD-PA	36.11		<0.001
SL (min)	Control vs. IBD	13.10	1.08	<0.001
	Control vs. IBD-PA	18.55		<0.001
	IBD vs. IBD-PA	5.44		<0.001
SE (%)	Control vs. IBD	9.00	0.85	<0.001
	Control vs. IBD-PA	15.78		<0.001
	IBD vs. IBD-PA	6.78		<0.001
Number of WASO	Control vs. IBD	0.96	0.11	<0.001
	Control vs. IBD-PA	2.39		<0.001
	IBD vs. IBD-PA	1.42		<0.001
Time of WASO	Control vs. IBD	45.75	1.53	<0.001
	Control vs. IBD-PA	38.91		<0.001
	IBD vs. IBD-PA	6.84		<0.001
REM (min)	Control vs. IBD	19.59	0.90	<0.001
	Control vs. IBD-PA	27.79		<0.001
	IBD vs. IBD-PA	8.20		<0.001
S1	Control vs. IBD	18.12	0.68	<0.001
	Control vs. IBD-PA	14.25		<0.001
	IBD vs. IBD-PA	3.86		<0.001
S2	Control vs. IBD	10.61	1.98	<0.001
	Control vs. IBD-PA	14.66		<0.001
	IBD vs. IBD-PA	4.05		<0.001
S3+S4	Control vs.IBD	40.20	1.83	<0.001
	Control vs. IBD-PA	60.50		<0.001
	IBD vs. IBD-PA	20.30		<0.001

### Comparison of PQSI Indices of the Three Groups

The PQSI data of three groups were analyzed by variance analysis and LSD test. Variance analysis results showed that there were significant differences in PSQI score, sleep quality, sleep latency, sleep duration, habitual sleep efficiency, sleep disturbances, use of sleeping medications and daytime dysfunction among the three groups (*P* < 0.001). The results are given in Table 4.

**Table 4:** Variance analysis results of the differences between IBD group, IBD-PA group and control group in PSQI sleep parameters.

**PSQI indexes**	**Control group**	**IBD group**	**IBD-PA group**	***F***	***P***
	***n* = 120**	***n* = 120**	***n* = 120**		
PSQI score	5.81 ± 1.87	9.88 ± 2.07	12.09 ± 2.32	278.14	<0.001
Sleep quality	0.88 ± 0.98	1.63 ± 0.85	1.70 ± 0.88	30.49	<0.001
Sleep latency	0.96 ± 0.96	1.44 ± 0.82	2.03 ± 0.81	45.38	<0.001
Sleep duration	0.97 ± 0.83	1.58 ± 0.78	1.88 ± 0.79	40.79	<0.001
Habitual sleep efficiency	0.90 ± 0.82	1.66 ± 0.79	2.02 ± 0.79	60.61	<0.001
Sleep disturbances	0.97 ± 0.79	1.65 ± 0.78	2.08 ± 0.82	60.69	<0.001
Use of sleeping medications	0.11 ± 0.36	0.28 ± 0.58	0.35 ± 0.92	4.48	<0.001
Daytime dysfunction	1.03 ± 0.94	1.62 ± 0.79	2.03 ± 0.81	41.06	<0.001

LSD test results were showed in Table 5. Compared with the control group, the indexes of IBD group and IBD-PA group were significantly different. Scores in all the seven dimensions and the total score of IBD group and IBD-PA group were significantly higher than the control group. In addition, IBD-PA group scored significantly higher than IBD group in sleep latency, sleep duration, habitual sleep efficiency, sleep disturbances, daytime dysfunction, and total score. But there was no significant difference between them in self-evaluation sleep quality and use of sleeping medications. According to the scoring rules of the PQSI scale and basing on the total scores, the above results illustrate that compared with the control group, IBD group and IBD-PA group showed worse sleep quality. Compared with the IBD group, the sleep quality of IBD-PA group was even worse.

**Table 5:** LSD test results of the differences between IBD group, IBD-PA group, and control group in PSQI sleep parameters.

**PSQI indexes**	**Group**	**Mean deviation**	**SD**	***P***
PSQI score	Control vs. IBD	4.08	0.27	<0.001
	Control vs. IBD-PA	6.28		<0.001
	IBD vs. IBD-PA	2.21		<0.001
Sleep quality	Control vs. IBD	0.75	0.12	<0.001
	Control vs. IBD-PA	0.83		<0.001
	IBD vs. IBD-PA	0.08		0.521
Sleep latency	Control vs. IBD	0.48	0.11	<0.001
	Control vs. IBD-PA	1.07		<0.001
	IBD vs. IBD-PA	0.58		<0.001
Sleep duration	Control vs. IBD	0.62	0.10	<0.001
	Control vs. IBD-PA	0.92		<0.001
	IBD vs. IBD-PA	0.30		0.004
Habitual sleep efficiency	Control vs. IBD	0.76	0.10	<0.001
	Control vs. IBD-PA	1.12		<0.001
	IBD vs. IBD-PA	0.36		0.001
Sleep disturbances	Control vs. IBD	0.71	0.10	<0.001
	Control vs. IBD-PA	1.12		<0.001
	IBD vs. IBD-PA	0.41		<0.001
Use of sleeping medications	Control vs. IBD	0.18	0.09	0.042
	Control vs. IBD-PA	0.25		0.004
	IBD vs. IBD-PA	0.08		0.382
Daytime dysfunction	Control vs.IBD	4.08	0.27	<0.001
	Control vs. IBD-PA	6.28		<0.001
	IBD vs. IBD-PA	2.21		<0.001

### PSG and PSQI Indices Comparison Between Active and Remission IBD-PA Patients

The PSG and PSQI indices of active and remission IBD-PA patients were compared. Results showed that for the PSG indices, the SE of active patients was lower than the remission patients (*P* < 0.001). The number of WASO of active patients was larger than the remission patients (*P* < 0.001). The S1 of active patients was longer (*P* = 0.008) and S3 + S4 was longer than the remission patients (*P* = 0.01). Moreover, the PSQI total score of active patients was higher than the remission patients (*P* = 0.021). The results are shown in Table 6.

**Table 6:** PSG and PSQI indices comparison between active and remission IBD-PA patients.

		**Active *n* = 39**	**Remission *n* = 81**	***t***	***P***
PSG	TST (min)	337.05 ± 17.11	343.22 ± 19.21	1.706	0.091
	SL (min)	40.58 ± 11.83	39.62 ± 11.65	0.421	0.675
	SE(%)	64.88 ± 7.90	73.20 ± 6.65	6.032	<0.001
	Number of WASO	3.82 ± 0.56	3.11 ± 0.89	4.561	<0.001
	Time of WASO	52.14 ± 10.20	49.96 ± 9.98	1.113	0.268
	REM (min)	56.12 ± 6.66	58.05 ± 8.12	1.289	0.200
	S1	37.87 ± 6.12	34.77 ± 5.78	2.700	0.008
	S2	199.23 ± 10.98	201.76 ± 12.85	1.057	0.293
	S3+S4	43.92 ± 9.52	48.85 ± 9.64	2.634	0.010
PSQI	PSQI score	12.82 ± 2.25	11.74 ± 2.41	2.348	0.021
	Sleep quality	1.75 ± 0.91	1.68 ± 0.87	0.407	0.685
	Sleep latency	2.11 ± 0.75	1.99 ± 0.82	0.771	0.442
	Sleep duration	2.02 ± 0.66	1.81 ± 0.85	1.357	0.177
	Habitual sleep efficiency	2.18 ± 0.63	1.94 ± 0.87	1.538	0.127
	Sleep disturbances	2.23 ± 0.91	2.01 ± 0.79	1.359	0.177
	Use of sleeping medications	0.38 ± 0.94	0.34 ± 0.89	0.226	0.821
	Daytime dysfunction	2.15 ± 0.76	1.97 ± 0.84	1.133	0.260

### The Correlation Between Disease Activity and Sleep Quality in IBD-PA Patients

The correlations between disease activity and sleep quality (total score of PSQI and sleep efficiency of PSG) of IBD-PA patients were analyzed. For the CD patients, there was a positive correlation between Harvey-Bradshaw indices and total score of PSQI (*r* = 0.528, *P* < 0.05), and a negative correlation between Harvey-Bradshaw indices and sleep efficiency of PSG (*r* = −0.478, *P* < 0.05). Similarly, for the UC patients, there was a positive correlation between MES and total score of PSQI (*r* = 0.673, *P* < 0.05), and a negative correlation between MES and sleep efficiency of PSG (*r* = −0.566, *P* < 0.05).

### The Effect of Demographic Characteristics and Disease Condition on the IBD-PA Patients' Sleep Efficiency of PSG

Univariate analysis was used to compare sleep efficiency of patients with different demographic characteristics and disease conditions. Table 7 presents the results. Then using sleep efficiency as dependent variable, the data of IBD-PA patients were analyzed by multiple linear regression. The results showed that abdominal pain, joint pain, depression, and C-reactive protein were the influencing factors of sleep efficiency. The results are shown in Table 8.

**Table 7:** Comparison of sleep efficiency of patients with different demographic characteristics and diseases situation.

	***n***	**%**	**Score**	**F/t**	***P***
**Gender**				1.292	0.858
Male	64	53.33	69.75 ± 7.78		
Female	56	46.67	69.57 ± 8.88		
**Marriage**				2.752	0.068
Unmarried	19	15.83	66.74 ± 7.19		
Married	94	78.33	70.57 ± 8.12		
Divorced or widowed	7	5.83	65.43 ± 11.03		
**Educational background**				1.096	0.354
Primary school or below	3	2.50	70.33 ± 4.04		
Junior middle school	9	7.50	68.44 ± 8.08		
Senior middle school	17	14.17	66.53 ± 10.24		
College or above	91	75.83	70.35 ± 7.95		
**Work status**				1.387	0.178
Employed	105	87.5	69.95 ± 8.58		
Unemployed	15	12.5	67.67 ± 5.50		
**Course of disease**				−1.031	0.304
≤ 5 years	68	56.67	68.99 ± 8.20		
>5 years	52	43.33	70.56 ± 8.37		
**Disease species**				−1.798	0.075
CD	17	14.17	66.35 ± 7.53		
UC	103	85.83	70.21 ± 8.30		
**Nocturnal digestive tract symptoms**				0.081	0.935
Yes	34	28.33	69.76 ± 9.25		
No	86	71.67	69.63 ± 7.92		
**Hormone use**				−2.499	0.014
Yes	33	27.5	66.67 ± 7.83		
No	87	72.5	70.80 ± 8.20		
**Sleeping pills use**				−1.996	0.048
Yes	17	14.17	66.0 ± 06.55		
No	103	85.83	70.27 ± 8.40		
**Disease activity**				−6.255	<0.001
Active	51	42.5	64.88 ± 7.90		
Remission	69	57.5	73.20 ± 6.65		
**Perianal disease**				−4.911	<0.001
Yes	22	18.33	62.5 ± 8.35		
No	98	81.67	71.28 ± 7.39		
**Disease extension**				0.529	0.786
Proctitis - UC	15	12.5	70.13 ± 9.03		
Left colitis-UC	77	64.17	70.17 ± 8.12		
Pancolitis-UC	11	9.17	70.64 ± 9.35		
Ileitis-CD	5	4.17	66.8 ± 9.36		
Ileocolitis-CD	6	5	66.5 ± 8.31		
Colitis-CD	4	3.33	65.75 ± 8.18		
Upper GI involvement-CD	2	1.67	66 ± 2.83		

**Table 8:** Influencing factors of objective sleep efficiency in patients with IBD-PA.

	**β**	**Standard error**	***t***	***P***	**95%CI**
Constant term	90.989	2.299	39.576	<0.001	86.433, 95.545
Age	0.045	0.029	1.556	0.122	−0.012, 0.102
**Disease activity (Reference group: Active stage)**					
Remission stage	−0.917	0.502	−1.827	0.070	−1.912, 0.078
**Perianal disease (Reference group: Yes)**					
No	−0.908	0.582	−1.558	0.122	−2.062, 0.247
Joint pain index	−0.763	0.124	−6.170	<0.001	−1.008, −0.518
**Hormone use (Reference group: Yes)**					
No	−0.298	0.468	−0.636	0.526	−1.225, 0.629
**Sleeping pills use (Reference group: Yes)**					
No	0.749	0.558	1.341	0.183	−0.358, 1.855
SDS score	−0.088	0.021	−4.301	<0.001	−0.129, −0.048
Abdominal pain	−1.052	0.382	−2.750	0.007	−1.811, −0.294
CRP	−0.610	0.050	−12.130	<0.001	−0.709, −0.510

## Discussion

According to the results, the objective sleep parameters and subjective self-assessment of sleep quality between the control group, IBD group, and IBD-PA group were significantly different.

First, compared with the control group, patients in IBD group showed poorer sleep quality in both PSG data and PSQI. Sleep disorder is a common problem in patients with IBD. This result was similar to those of previous studies. For example, Uemura et al. reported the prevalence of sleep disturbances was 44.1% in Japanese IBD patients (23). In a study from China, 55.8% IBD patients reported poor sleep (16). There are few studies on PSG in IBD patients. Compared with Keefer et al. research, some results are similar. For example, they found that IBD patients had less sleep efficiency and TST than the control group, which are similar with ours. In their research, the average sleep efficiency of IBD patients was 84.44 ± 5.06 while it was 78.0 ± 13.43 in control group. The average TST of IBD group was 418.55 ± 55.67, while in the control group the average TST was 464.18 ± 19.26. The sleep efficiency were higher and TST were longer than those in our study. Unlike our findings, there was no significant difference in other indicators between the two groups (24). Bar-Gil Shitrit et al. found a significantly less REM sleep was noted in the inactive IBD patient group vs. control and light sleep percentage and REM latency were also longer in the IBD group. All other sleep parameters including number of wakes, sleep latency, were similar in both groups (25). Sleep problems of patients include short sleep time, difficulty in falling asleep, increased number of WASO, etc (26). Summarizing their research, the factors affecting sleep quality of IBD patients include disease active period, unemployment, female, hormone therapy, restless legs syndrome, fatigue, anxiety, inflammatory factors, and so on (16, 27–29).

Second, when it comes to comparing the sleep quality between the control group and IBD-PA group, results showed the IBD-PA group was poorer. In previous studies, there were more questionnaire surveys on sleep of IBD patients, but we haven't found any studies focused on sleep of IBD-PA patients. In addition to the possible causes of sleep disorders in patients with IBD in the first part, these patients not only suffer from digestive tract symptoms, but also experience joint pain and limited movement, which may seriously affect their sleep.

Third, compared with IBD patients, the sleep quality of IBD-PA patients was even worse. Sleep status of patients with IBD-PA is the focus of this study. As to the objective sleep parameters, the patients showed very low sleep efficiency and very short sleep time, which indicated that the patients' sleep problems were serious and should be paid great attention to. In terms of sleep structure, the patients' slow wave sleep (deep sleep, S3 + S4) time is shorter. Deep sleep is associated with cognition and helps patients regain energy (30). The rapid eye movement sleep of patients is also short, which has a negative effect on the mental health of patients (31). Therefore, attention should be paid to patients' mental health, such as cognition. As to the subjective assessment results, patients' with IBD-PA reported higher scores in all the 7 items and the total score of PSQI than IBD group, which indicated their subjective sleep quality was worse.

In this study, it was found that sleep was in relation to disease activity in IBD-PA patients. Active patients had worse sleep than remission patients. This was the same as other studies. For example, Graff et al. conducted a PSQI survey of 318 adult IBD patients and found that 82% of active IBD patients had sleep disorder, while 51% of remission IBD patients had sleep disorder (32). Ali et al. confirmed that 100% of patients in active stage of disease had sleep disorders, while only 54% of patients in remission stage had sleep disorders (11). This is because the physical and mental health of patients in the active stage of disease is worse, which affects patients' sleep. In addition, cytokines also affect sleep. In addition, the levels of cytokines were different between active patients and remission patients. Cytokines interact with sleep. For example, IL-1 can regulate sleep through serum receptor pathways. Low levels of IL-1 can promote NREM, while high levels of IL-1 can inhibit NREM (33).

In order to explain why IBD-PA patients had the poorest sleep quality than the IBD patients and the control group, regression, and correlation analysis were used to explore the influencing factors of objective sleep efficiency. Results showed that abdominal pain, joint pain, depression, and C-reactive protein were the influencing factors of sleep efficiency in patients with IBD-PA. This study found that patients with gastrointestinal symptoms, such as abdominal pain, reduced their sleep quality. This is the same as previous research results. For example, Pirinen et al. found that sleep disorders were associated with clinical symptoms. In adolescent IBD patients, 41% of patients with severe clinical symptoms and 22% of patients with mild clinical symptoms had sleep disorders (34). Joint pain severely affects the patient's sleep. Patients' with higher pain index got lower sleep efficiency because pain causes frequent awakening during sleep. Depression is also an important factor in sleep, which is the same as other studies (35, 36). IBD patients often have negative emotions such as anxiety and depression because the course of illness is long and repeated, which seriously affects work and life, and patients perceive the uncertainty of illness (37). Moreover, emotions affect the outcome of disease and the quality of life of patients (38). Patients with IBD-PA are more likely to have mental health problems because they have more joint symptoms on this basis. Pain, negative emotions and sleep disorders often form a vicious circle. This suggests that pain management and psychological intervention should be strengthened in patients with IBD-PA (39). In addition, CRP, a marker of inflammation, was related to sleep. In inflammatory diseases, the body produces NF-kB, which affects sleep rhythm (40). Higher CRP means more inflammation, so sleep is more affected. Understanding these factors is helpful for targeted sleep intervention.

## Limitations

There are limitations of our present study. First, there are many factors affecting the patients' sleep quality, including hormone level, sport, nutritional status, intestinal flora, etc. In the present study, we incorporated limited factors. Clearly, it is difficult and unpractical to control for all these factors in a study. We can only control a certain number of the factors. Second, in the selection of the object, the sample size was small because the total number of such patients is relatively small. Third, this was a cross-sectional study that only measured patients' indices at a single time point. Therefore, it has a certain contingency; long-term intervention studies and cohort studies need to be conducted in the future.

## Conclusion

Generally speaking, the objective and subjective sleep quality of patients with IBD were poor, and these of IBD-PA patients were even worse. Specifically, on objective sleep quality, the TST, sleep efficiency, REM time, S2, S3 + S4 of IBD, and IBD-PA group were shorter than the control group. However, the sleep latency, number of WASO, time of WASO and S1 of IBD and IBD-PA group were longer or more than the control group. The above sleep problems of IBD-PA group were more serious than IBD group. On subjective sleep quality, all the 7 dimension scores and the total score of PSQI in IBD group and IBD-PA group were higher than the control group. IBD-PA group scored significantly higher than IBD group in sleep latency, sleep duration, habitual sleep efficiency, sleep disturbances, daytime dysfunction, and total score. The influencing factors of IBD-PA patients' sleep quality were hormone use, nocturnal gastrointestinal symptoms, joint pain, depression, and disease activity. Therefore, sleep management should be integrated into IBD management.

## Data Availability Statement

The datasets generated for this study are available on request to the corresponding author.

## Ethics Statement

This study was carried out in accordance with the recommendations of Wuhan University School of Medicine Ethics Committee (No. 20161101) with written informed consent from all subjects. All subjects gave written informed consent in accordance with the Declaration of Helsinki. The protocol was approved by the Wuhan University School of Medicine Ethics Committee.

## Author Contributions

NY designed the research. YZ and BP were responsible for drafting the manuscript and collected the data. YL and XC analyzed the data. YZ, NY, and XL reviewed and revised the manuscript. XX reviewed the manuscript. All authors approved this version to be published.

### Conflict of Interest Statement

The authors declare that the research was conducted in the absence of any commercial or financial relationships that could be construed as a potential conflict of interest.
